# Examining the impact of health research facilitated by small peer-reviewed research operating grants in a women's and children's health centre

**DOI:** 10.1186/1756-0500-3-107

**Published:** 2010-04-20

**Authors:** Andrew J Caddell, Jill E Hatchette, Patrick J McGrath

**Affiliations:** 1 Research Services, IWK Health Centre, 5850/5980 University Avenue, PO Box 9700, Halifax, NS, B3K 6R8, Canada; 2 Department of Pediatrics, Psychiatry and Psychology, Dalhousie University, Halifax, NS, B3H 3J5, Canada

## Abstract

**Background:**

There has been limited research on the impact of research funding for small, institutional grants. The IWK Health Centre, a children and women's hospital in Maritime Canada, provides small amounts (up to $15,000) of research funding for staff and trainees at all levels of experience through its Research Operating Grants. These grants are rigorously peer-reviewed. To evaluate the impact of these grants, an assessment was completed of several different areas of impact.

**Findings:**

An online questionnaire was sent to 64 Principal Investigators and Co-Investigators from Research Operating Grants awarded from 2004 to 2006. The questionnaire was designed to assess five areas of potential impact: (1) research, (2) policy, (3) practice, (4) society and (5) personal. Research impact reported by participants included publications (72%), presentations (82%) and knowledge transfer beyond the traditional formats (51%). Practice impact was reported by 67% of participants, policy impact by 15% and societal impact by 18%. All participants reported personal impact.

**Conclusions:**

Small research grants yield similar impacts to relatively large research grants. Regardless of the total amount of research funds awarded, rigorously peer-reviewed research projects have the potential for significant impact at the level of knowledge transfer and changes in clinical practice and policy. Additional findings in the present research indicate that small awards have the potential to have significant impact on the individual grant holder across a variety of capacity building variables. These personal impacts are particularly noteworthy in the context of developing the research programs of novice researchers.

## Background

Although health research focuses on the development of evidence, there has been surprisingly little effort to evaluate the effects of research and research funding programs. Some research program evaluations of agencies such as the NIH[1] and the Health and Health Research Council of New Zealand[2] have relied on bibliometric methods to review their granting programs. These reviews typically consist of recording publications per funded project and using this information to compare the number of papers published among comparable institutes or grants[1,2]. Although useful, these evaluations are limited in scope, and do not address the multidimensional aspects of research impact[3].

Research frameworks have been developed by several groups[3,4] and institutions[5] as a means for evaluating of assessing a range of impacts resulting from health services research. health research. The Research Impact Framework[3] identifies four areas of impact: Research-related impacts include traditional measures of research success (publications, conferences, patents and awards) as well impacts on the research field itself through communication, knowledge translation and research networks; policy impacts include research that informs and influences policy; service impacts include improved methods and cost-effectiveness of delivering quality health-care; societal impacts include outcomes that affect the population beyond the research world. A clear research impact framework can contribute greatly to the evaluation of research grant programs by identifying the areas of potential impact and illustrating the relationships between them.

Agencies such as The Health and Health Services Research Fund of Hong Kong[6], Public Health Research and Development Committee of Australia[7] and the Alberta Heritage Fund for Medical Research[8] have employed similar frameworks in the evaluation of granting programs to demonstrate significant policy, service and research impacts[6-8]. However, these frameworks are typically geared toward large grants with equally large outcomes and are not easily transferable to the evaluation of smaller scale research projects designed to build research capacity. To our knowledge, there is no literature that assesses the impact of very small grants or suggests a framework within which to evaluate the impact of very small grants.

The IWK Health Centre is a women and children's academic health sciences centre, affiliated with Dalhousie University, serving Maritime Canada. The IWK has a vigorous research culture that thrives across disciplines and levels of expertise. Through the IWK's Research Services Department, a variety of granting opportunities are available within the research award program. The goal of this program is to promote the development of high quality research, consistent with the mission of the health centre. These grants are designed to support research that can be completed within a proposed budget or provide seed funding for the development of larger proposals for external funding. The grants include Category A grants funding up to ($4,000.00) and Category B grants (funding up to $15,000.00). These awards undergo rigorous peer review by the IWK Scientific Review Committee. Since 2002, over $800,000 has been awarded through 165 Category A and B grants, mostly to novice and junior researchers.

The research impact literature has no known research addressing the impact of very small research grants. Nor are there any suitable frameworks in which to measure the impact of very small grants. The purpose of the present study was to describe the impact of health research facilitated by Research Operating Grants at the IWK Health Centre within the context of a relevant research impact framework. To this end, an adaptation of the Research Impact Framework[3] was used to assess the dimensions most applicable to the impact of very small grants.

### Design

Retrospective on-line survey conducted between June 6, 2008 and August 22, 2008.

### Participants

Principal Investigators of Research Operating Grants (valued at under $15,000) from the IWK Health Centre awarded between the years 2004-2006. Multiple IWK grant holders were queried on only one grant randomly selected by the investigators. After exclusions for multiple grants, 64 eligible grant holders were invited to participate. Seven investigators declined to participate and 18 failed to complete the questionnaire, leaving a final group of 39 (61%).

Measures: The Research Impact Assessment Questionnaire [additional file 1] was adapted from the Research Impact Framework[3] and included 5 subsections designed to measure various impacts of the research. Subsections included: (1) research (impact on research itself), (2) policy (impact and influence on policy), (3) practice (impact and influence on medical practice), (4) society (impact on society) and (5) personal (measure of self or career development). Additional sections were included for demographic information, difficulties encountered during the research and future plans for research.

The survey was hosted on a secure web server maintained by Dalhousie University. The Opinio Web Server uses Opinio survey software which allows researcher to produce and publish online surveys. Questionnaire items included multiple choice, short answer and yes/no questions. For almost all of the questions, a comment box was provided for participants to elaborate on their responses.

### Procedure

Potential participants were identified and contacted using information provided in the IWK Research Services' database. Contact and consent were completed by email. Participants were provided a randomly generated identification number to access the questionnaire.

Reminders were sent via email to participants who had not completed the questionnaire after 2 weeks and again after 4 weeks. After a further 2 weeks of inactivity following the second reminder sent to Principal Investigators, Co-Investigators were invited to complete the questionnaire instead.

## Results

### Demographics

The response rate for this study was 61%. Data were collected from 36 Principal Investigators and 3 Co-Investigator (n = 39; 28 female). Of the 39 grants, 31 were Category A grants and 8 were Category B grants. Given the small number of Category B grants, data were combined since comparisons would not yield meaningful information. Fifty-seven percent of participants held their primary affiliation at the IWK Health Centre; 43% were cross-appointed from Dalhousie University. Fifty-six percent of participants held PhDs or MDs and the range of research experience across participants were quite varied (see Table 1).

**Table 1:** Research experience of participants (n = 39).

*Research experience (years)*	*% of participants*
<3	26%
3-6	36%
7 - 10	20%

10 +	18%

Although a wide range of disciplines were represented across grants, Nursing (26%) and Psychology (31%) accounted for over 50% of the grants held.

### Research Impact

Close to three-quarters of the participants reported research publications in the form of either peer-reviewed journals or abstracts (See Table 2). A large percentage of participants also indicated that they had presented their research at scientific conferences.

**Table 2:** Research impacts associated with small grant research.

Research publications	72% (n = 28; range = 0 - 4)
Peer-reviewed journal	45% (n = 12; range = 1 - 2)
Abstract	56% (n = 16; range = 1 - 3)
Presentation at scientific conferences	82% (n = 32)
Knowledge translation (other)	51% (n = 20)

Collaborations with other centres	49% (n = 19)
Existing^1^	26% (n = 5)
New^2^	74% (n = 14)

1. Refers to collaborations that were already in place prior to funding

2. Refers to collaborations that developed as a result of funding.

The total number of conferences that grant-holders presented their finding at was sixty-one. Aside from these traditional forms of knowledge transfer, over half of the respondents reported knowledge translation to parents, practice groups, educators and professional associations. Demonstrating the collaborative nature of research, almost half of the participants indicated that their projects relied on established or new scientific collaborations with other scientific or academic centres.

### Policy, Practice and Society Impact

Six participants reported that their projects had positive impacts on policy change either within or external to the health centre (See Table 3). Although only one-third of projects resulted in a change in clinical practice, more than half of the participants indicated their research informed clinical practice by providing clinicians and staff with a broader clinical understanding and increasing awareness.

**Table 3:** Policy, practice and society impacts associated with small grant research.

Policy effect	16% (n = 6)
Within health centre	8% (n = 3)
Beyond health centre	8% (n = 3)
Changes in clinical practice	32% (n = 12)
Informing clinical practice	55% (n = 21)

Improving quality of care	46% (n = 18)
Patient safety	18% (n = 7)
Cost effectiveness	10% (n = 4)
Access to care	5% (n = 2)
Adaptation of health services	13% (n = 5)

Almost half of participants reported that, on some level, their research improved the quality of care delivered through patient safety, cost-effectiveness, access to care or adaptation of health services. Even though these impacts were seen primarily at the level of the health centre, continued implementation of change and future directions of these research projects may see larger societal impacts. None the less, approximately 18% of participants reported initiatives to inform the public of their research through press releases, public talks and workshops to name a few.

### Personal Impacts

The most significant personal impacts reported by participants included further research development and mentorship (See Table 4). Research development included completing graduate theses, refining specific research skills like data analysis and appreciating the complexities of good science. Although 15% of participants pursued additional formal education because of the research, mentorship appeared to be more important in the research development process. Participants reported the benefits of mentoring clinical staff and undergraduate students as well as being mentored in areas of research that were previously new to them.

**Table 4:** Personal impacts associated with small grant research.

Pursuit of additional education	15% (n = 6)
Mentorship of PI by a senior researcher	60% (n = 23)
Mentorship by PI of a junior researcher	67% (n = 26)
Further development of researcher goals	77% (n = 30)

Improvement of research skills	87% (n = 34)

### Relationships between impacts

Although many of the relationships that were explored between impacts were not statistically significant, a few relationships were particularly noteworthy.

For example, there was a significant relationship between the investigators research experience and the number of conferences that the study was presented at (χ^2 ^(4), N = 39) = 10.06, p < .05). Research experience was also related to mentoring (χ^2 ^(4), N = 39) = 9.3, p < .05) in that researchers with very little experience indicated that they had been mentored by a more senior researcher. In terms of knowledge transfer, there was a significant relationship between presentations given at conferences and research findings informing clinical practice (χ^2 ^(3), N = 39) = 14.29, p < .05).

## Discussion

The results from this research evaluation demonstrate the effectiveness of small peer reviewed operating grants such as the IWK Research Services' Operating Grants Program. Despite the relative small funding amount, impacts comparable to larger grants were clearly apparent. For example, when considering the more traditional forms of knowledge transfer such as publishing in peer reviewed journals, the number of publications reported by participants (72%) was similar to those reported from granting agencies providing significantly larger funds. For example, Kwan et al. reported that 86.5% of projects funded by the Health and Health Services Research Fund in Hong Kong yielded peer-reviewed publications while Shah and Ward reported 82% of Australian Public Health Research and development Committee grants produced peer-reviewed publications [6,7]. Many of the participants also reported additional forms of knowledge transfer, typically consisting of presentations to research teams, trainees or clinical staff at the IWK Health Centre, other hospitals or a local university. Presentations to the community were also common. Similar to knowledge transfer, various policy and practice impacts resulted from the research conducted by the participants. These policy and practice impacts ranged in scope from local to national and included: changes to policy regarding newborn screening processes to minimize false positives for genetic testing, policy changes regarding immunization against influenza for pregnant women and implementing disciplinary tracking systems for the Department of Education. Societal impact was seen through the dissemination of research results to the public, whether through popular press, conferences or other methods.

A unique focal point to this review was the area of personal impact. Nearly all of the participants reported a positive experience and felt that this research improved their research skills and developed their program of research. A positive impact at the level of the research is a fundamental component of ensuring that novice and junior researchers continue to pursue research opportunities. Perhaps most notable of the personal impacts was the opportunity to mentor and be mentored. Mentorship is essential to building the research careers of novice investigators by providing support and expertise.

Relationships between impacts demonstrate the interconnectedness of the areas of potential impact. The associations found between practice impacts and presentations, demonstrate a link between knowledge transfer and effects on practice. Similarly, the association between research experience and conference presentations indicates that as investigators become more experienced, they engage in more knowledge transfer activities.

The present study however, is not without limitations. The participation rate, although reasonable, may have contributed to bias. Investigators who had projects with little impact may have decided not to participate. The small sample size limited the use of inferential statistics that might provide more insight into the relationships between variables. Finally, the exclusive reliance on self report by the participants on the impact of their research may have biased the results.

## Conclusion

The foundation of excellence in health service delivery is the evidence that informs clinical practice and policy change. It is essential that academic health centres engage actively in ensuring that a culture of research inquiry is maintained and that support is available to those researchers that may ultimately contribute to the excellence in health care. Typically, the purpose of small institutional grants is to provide the seed funding that will support new researchers in the development of their programs of research. The findings from this study indicate that the grants awarded through the IWK Health Centre provide researchers with the support they need to carry out their research, engage in effective knowledge translation and affect policy and clinical practice. Perhaps more importantly are the personal impacts that small-scale funding has on researchers such as the research capacity that is built through new collaborations, mentorship, and the confidence and skill to pursue further research endeavours.

## Competing interests

Jill Hatchette and Patrick McGrath have both been awarded Category A grants as Principal Investigators and Co-investigators. Jill Hatchette was included as one of the participants in this study.

## Authors' contributions

AC: Conducted the background literature review, participated in study design, collected all data, performed data analyses and drafted the manuscript. JH: Participated in study design, reviewed data analysis, prepared final manuscript.

PM: Conceived of study, participated in study design, and reviewed manuscript drafts.

All authors read and approved the final manuscript.

## Supplementary Material

Additional file 1 **IWK Research Impact Assessment Questionnaire**. The Research Impact Assessment Questionnaire is composed of 5 subsections designed to measure various impacts of research. Subsections included: (1) research (impact on research itself), (2) policy (impact and influence on policy), (3) practice (impact and influence on medical practice), (4) society (impact on society) and (5) personal (measure of self or career development). The questionnaire also included sections designed to collect demographic information, information about difficulties encountered during the research and future plans for research. Click here for file
