# Supplementary material for: Examining the impact of health research facilitated by small peer-reviewed research operating grants in a women's and children's health centre
Source: BMC Res Notes. 2010 Apr 20;3:107. doi: 10.1186/1756-0500-3-107 (PMC2873346; doi:10.1186/1756-0500-3-107)
Supplement: Additional file 1 — IWK Research Impact Assessment Questionnaire. The Research Impact Assessment Questionnaire is composed of 5 subsections designed to measure various impacts of research. Subsections included: (1) research (impact on research itself), (2) policy (impact and influence on policy), (3) practice (impact and influence on medical practice), (4) society (impact on society) and (5) personal (measure of self or career development). The questionnaire also included sections designed to collect demographic information, information about difficulties encountered during the research and future plans for research. [file 1756-0500-3-107-S1.DOC]

**IWK Research Impact Assessment Questionnaire**

# I. Introduction

# These questions were designed to gather information about the health and personal impacts of research aided by IWK category A and B grants. The results of this questionnaire may be useful in facilitating a more productive research environment.

# II. Demographic Information

1. What is your assigned participant identification number? ____________
2. Sex of the PI: Male Female
3. Highest qualification of the PI:

| Bachelor’s | Master’s | PhD | MD | Other (specify) |
| --- | --- | --- | --- | --- |
|  |  |  |  |  _________ |

1. What is the discipline of the PI? ____________________________________
2. Primary affiliation at the time the research was funded:

| IWK Health Centre | Dalhousie University | CDHA | Other (specify________) |
| --- | --- | --- | --- |
|  |  |  |  |

1. Secondary affiliation at the time the research was funded:

| IWK Health Centre | Dalhousie University | CDHA | Other (specify________) | Not applicable |
| --- | --- | --- | --- | --- |
|  |  |  |  |  |

1. Position at academic institution:

| Instructor/ Lecturer | Assistant Professor | Associate Professor | Full Professor | Other (specify________) | Not applicable |
| --- | --- | --- | --- | --- | --- |
|  |  |  |  |  |  |

1. Research experience at the time the research project was funded (in years):

|   None |   Less than 3 |   3-6 |   7-10 |   More than 10 |
| --- | --- | --- | --- | --- |

# III. Research-related Questions

All further questions relate to Category A or B grant indicated. Please refer to the initial email to help you in these questions.

1. Grant type:  
   Category A B
2. Was the research project:
    
   Single centered Multi-centered
3. a) Had you received funding from the IWK Health Centre previous to this grant?
    
   Yes No

   b) If yes, please list the most recent funding previous to this grant.

| Grant Amount | Category A | Category B | Other |
| --- | --- | --- | --- |
|  |  |  |  Please specify __________ |
|  |  |  |  Please specify __________ |
|  |  |  |  Please specify __________ |
|  | **** | **** | **** Please specify __________ |
|  | **** | **** | **** Please specify __________ |

1. a) Was the research project completed?
    
   Yes No

   b) If no, please explain why.
   ______________________________________________________________________________________________________________________________________________________________________________________________________
2. a) Was the project funded by additional sources from the onset of the project?
    
   Yes No

   b) If yes, please list.

| Funding Agency | Funding amount |
| --- | --- |
|  |  |
|  |  |
|  |  |
|  |  |
|  |  |

1. Did you experience significant difficulties with any of the following during the research process:

|  | Yes | No | Not Applicable |
| --- | --- | --- | --- |
| Obtaining ethical approval? |  |  |  |
| If yes, please explain. ____________________________________________________________________________________________________________________________________________________________________________________ | | | |
|  | Yes | No | Not Applicable |
| Accessing funds? (e.g. setting up accounts, billing, reimbursement of participants) |  |  |  |
| If yes, please explain. ____________________________________________________________________________________________________________________________________________________________________________________ | | | |
| Hiring staff? |  |  |  |
| If yes, please explain. ____________________________________________________________________________________________________________________________________________________________________________________ | | | |
| Recruiting participants |  |  |  |
| If yes, please explain. ____________________________________________________________________________________________________________________________________________________________________________________ | | | |
| Methodological problems |  |  |  |
| If yes, please explain. ____________________________________________________________________________________________________________________________________________________________________________________ | | | |
| Data analysis |  |  |  |
| If yes, please explain. ____________________________________________________________________________________________________________________________________________________________________________________ | | | |

If applicable, please identify and explain any additional difficulties encountered during the research procedure.
_______________________________________________________________________________________________________________________________________________________________________________________________________________

1. a) Were the results from this research published:

|  | Yes | No | If yes, how many publications |
| --- | --- | --- | --- |
| In a peer-reviewed journal? |  |  |  |
| As an abstract? |  |  |  |
| As a book chapter? |  |  |  |
| As a technical report? |  |  |  |

b) If any other types of publications resulted, please the type and number.
______________________________________________________________________________________________________________________________________________________________________________________________________

1. If the results of this research were published in a peer-reviewed journal, please list the journal name. (If the results of the research were published in more than one peer-reviewed journal, please list all journals.)

   ______________________________________________________________________________________________________________________________________________________________________________________________________

1. Were the results of the research presented at:

|  |  |  | If yes, how were the results presented as: | |
| --- | --- | --- | --- | --- |
|  | Yes | No | Talk | Poster |
| A local scientific conference? |  |  |  |  |
| A regional scientific conference? |  |  |  |  |
| A national scientific conference? |  |  |  |  |
| An international scientific conference? |  |  |  |  |

1. Did any new patents result from this research?
    
   Yes No
2. a) Was there other knowledge transfer to scientific, professional or administrative groups resulting from this research? (This might include presentations to practice groups, distribution of summaries to administrators.)
    
   Yes No
   b) If yes, please explain.
   ______________________________________________________________________________________________________________________________________________________________________________________________________
3. a) Did you receive any awards because of this research?
    
   Yes No
   b) If yes, how many:

| Local awards? |  |
| --- | --- |
| Regional awards? |  |
| National awards? |  |
| International awards? |  |

1. a) Were scientific collaborations made within the IWK, Dalhousie or external centers (academic/health) as a result of obtaining this grant?
    
   Yes No

   b) If yes, were these collaborations previously existing, or the results of research?
    
   Existing Results of research
2. a) Did this category A or B grant result in additional funding for related research?
    
   Yes No

   b) If yes, please list.

| Funding agency | Amount of Funding |
| --- | --- |
|  |  |
|  |  |
|  |  |
|  |  |
|  |  |

# Part IV. Policy-related questions

# Please note that a policy is defined as a plan at any institution to influence decisions or actions.

1. a) Did the research affect any existing policies at the IWK?
      
   Yes No Don’t know Not Applicable

   b) If yes, please describe.
   ______________________________________________________________________________________________________________________________________________________________________________________________________
2. a) Were policies affected outside the IWK?
      
   Yes No Don’t know Not Applicable

   b) If yes, at what level.
       
   National Provincial Municipal Other Health Centre Don’t know

   c) Please describe.
   ______________________________________________________________________________________________________________________________________________________________________________________________________

# V. Practice-related questions

1. a) Did the research change clinical practice?
      
   Yes No Don’t know Not Applicable

   b) If yes, then how?
   ______________________________________________________________________________________________________________________________________________________________________________________________________

   c) Did the research inform clinical practices, even if it didn’t change practice?
      
   Yes No Don’t know Not Applicable

   d) If yes, please explain.
   ____________________________________________________________________________________________________________________________________
   __________________________________________________________________
2. Did the research result in improvements to the quality of care via:

|  | Yes | No |
| --- | --- | --- |
| Access to care. (E.g. for rural or high risk populations) |  |  |
| Patient safety. |  |  |
| Adapting health services to respond to population health needs. |  |  |
| Increased cost-effectiveness of health care delivery. |  |  |

Please explain all selected responses.
_______________________________________________________________________________________________________________________________________________________________________________________________________________

1. a) Were any new products developed as a result of this research?
    
   Yes No

   b) If yes, describe.
   ______________________________________________________________________________________________________________________________________________________________________________________________________

# VI. Societal-related questions

1. a) Were the research results disseminated to the public?
    
   Yes No

   b) If yes, how were the results disseminated? ______________________________________________________________________________________________________________________________________________________________________________________________________

   c) Who were the results disseminated to? ______________________________________________________________________________________________________________________________________________________________________________________________________
2. a) Did the research result in any other impact?
    
   Yes No

   b) If yes, please explain the impact.
   ______________________________________________________________________________________________________________________________________________________________________________________________________

# VII. Personal

1. a) Did the research cause you to pursue additional education?
    
   Yes No

   b) If yes, please list all additional education received.
   ______________________________________________________________________________________________________________________________________________________________________________________________________
2. Did the research allow you to:

|  | Yes | No |
| --- | --- | --- |
| a) be mentored? (E.g. by a senior researcher) |  |  |
| b) mentor someone else? (E.g. a student or trainee) |  |  |

1. a) Did this grant help strengthen your research skills?
    
   Yes No

   b) If yes, please explain.
   ____________________________________________________________________________________________________________________________________
   __________________________________________________________________
2. a) Did the research contribute to the development of your own program of research?
    
   Yes No

   b) If yes, please explain.
   ______________________________________________________________________________________________________________________________________________________________________________________________________
3. a) Was the research a positive experience?
    
   Yes No
   b) Please explain.
   ______________________________________________________________________________________________________________________________________________________________________________________________________

# VIII. Future Work

1. a) Will you seek funding from the IWK Health Centre in the future?
      
   Yes No Don’t know

   b) If no, please explain.
   ____________________________________________________________________________________________________________________________________
   __________________________________________________________________

1. a) Do you have any comments on the IWK Research Programs?
   ____________________________________________________________________________________________________________________________________
   __________________________________________________________________

   b) Are there any ways you feel the IWK Research Programs can be improved?
   ____________________________________________________________________________________________________________________________________
   __________________________________________________________________
2. Would you like to receive a summary of results, when available? (Please note that if do not wish to a summary of the results sent to you, the results summary will be posted online on the Research Services Website )

 
Yes No

b) Please provide an email address.
______________________________________________________________________________________________________________________________________________________________________________________________________

*Thank you, your contribution is appreciated.*
